# An Elevated Gap between Admission and A_1C_-Derived Average Glucose Levels Is Associated with Adverse Outcomes in Diabetic Patients with Pyogenic Liver Abscess

**DOI:** 10.1371/journal.pone.0064476

**Published:** 2013-05-28

**Authors:** Wen-I Liao, Wayne Huey-Herng Sheu, Wei-Chou Chang, Chin-Wang Hsu, Yu-Long Chen, Shih-Hung Tsai

**Affiliations:** 1 Department of Emergency Medicine, Tri-Service General Hospital, National Defense Medical Center, Taipei, Taiwan; 2 Division of Endocrinology and Metabolism, Department of Internal Medicine, Taichung Veterans General Hospital, Taichung, Taiwan; 3 Department of Radiology, Tri-Service General Hospital, National Defense Medical Center, Taipei, Taiwan; 4 Department of Emergency and Critical Care Medicine, Taipei Medical University-Wan Fang Hospital, Taipei, Taiwan; St. Vincent’s Institute, Australia

## Abstract

**Objectives:**

To assess whether chronic glycemic control and stress-induced hyperglycemia, determined by the gap between admission glucose levels and A_1C_-derived average glucose (ADAG) levels adversely affects outcomes in diabetic patients with pyogenic liver abscess (PLA).

**Methods:**

Clinical, laboratory, and multi-detector computed tomography (MDCT) findings of 329 PLA patients (2004–2010) were retrospectively reviewed. HbA_1C_ levels were used to determine long-term glycemic control status, which were then converted to estimated average glucose values. For the gap between admission glucose levels and ADAG levels, we used receiver operating characteristic (ROC) curve to determine the optimal cut-off values predicting adverse outcomes. Univariate and multivariate logistic regressions were used to identify predictors of adverse outcomes.

**Results:**

Diabetic PLA patients with poorer glycemic control had significantly higher *Klebsiella pneumoniae* (KP) infection rates, lower albumin levels, and longer hospital stays than those with suboptimal and good glycemic control. The ROC curve showed that a glycemic gap of 72 mg/dL was the optimal cut-off value for predicting adverse outcomes and showed a 22.3% relative increase in adverse outcomes compared with a glycemic gap<72 mg/dL. Multivariate analysis revealed that an elevated glycemic gap≥72 mg/dL was important predictor of adverse outcomes.

**Conclusions:**

A glycemic gap≥72 mg/dL, rather than admission hyperglycemia or chronic glycemic control, was significantly correlated with adverse outcomes in diabetic PLA patients. Poorer chronic glycemic control in diabetic PLA patients is associated with high incidence of KP infection, hypoalbuminemia and longer hospital stay.

## Introduction

Stress-induced hyperglycemia commonly occurs in patients with critical illness including trauma, burn injuries, surgeries, myocardial infarction, and sepsis [1]. Acute hyperglycemia on admission can be caused by the presence of excessive counter-regulatory hormones (glucagon, growth hormone, catecholamines, and glucocorticoids) and anti-inflammatory cytokines as well increased gluconeogenesis and hepatic insulin resistance. [1]–[3].

Elevated blood glucose levels may indicate a severe illness with an increased response to stress. However, paradox existed in the discordant findings in the correlation between hyperglycemia and adverse outcomes in acute-ill patients with or without preexisting diabetes. In nondiabetic patients with infections, admission hyperglycemia was associated with adverse outcomes with a J-shaped curve (i.e., patients with initial glucose levels <100 mg/dL and >200 mg/dL have worse outcomes compared with those normal glucose levels) [4]. Nonetheless, the association between admission hyperglycemia and acute illness in patients with diabetes remains to be controversial. [4]–[6]. Several studies argued against the association between admission hyperglycemia and adverse outcomes in infected diabetic patients [4], [7]. In diabetic patient suffering acute illness, the epiphenomenon of acute hyperglycemia in patients with acute illnesses can result from acute physiological stress, chronic baseline blood glucose levels or both [7].

Glycated hemoglobin (HbA_1c_) reflects long-term glycemic control over the preceding 2–3 months; it is a better index of overall glycemic exposure and is characterized by lower biological variability, no fasting requirement, relative stability at room temperature, and is relatively unaffected by acute stress or sepsis [8]. An international multicenter A_1C_-derived average glucose (ADAG) study demonstrated a strong correlation between HbA_1C_ and long-term mean plasma glucose levels in the preceding 3 months, which allows estimation of long-term average glucose levels using HbA_1C_ values [9]. We speculated that elevated admission hyperglycemia in infected diabetic patients may reflect stress-induced hyperglycemia, a consequence of long-term poor glycemic control or in combination. This method allowed us to test the association between acute stress induced hyperglycemia by eliminating the influence of chronic hyperglycemia on the admission hyperglycemia in patient with pre-existing diabetes.

We hypothesize that the gap between admission hyperglycemia and the estimated long term average glucose levels (eAG) could represent stress-induced glycemic deterioration more properly than admission hyperglycemia alone among diabetic patients. This method may aid in investigating the sepsis severity in diabetic patients. To test this hypothesis, we conducted the present study on a well-known diabetic patient prone infection- pyogenic liver abscess (PLA) [10]–[13].

The purpose of this study was to clarify whether the gap between admission glucose levels and eAG derived from admission HbA_1C_ levels could predict adverse outcomes in diabetic PLA patients. In addition, the relationship between long-term glycemic control and clinical outcomes in diabetic PLA patients were analyzed.

## Materials and Methods

### Patients

This retrospective study was conducted at a tertiary referral medical center in northern Taiwan and was approved by the institutional review board for human investigations. The requirement for written informed consent from the patients was waived because patient anonymity was strictly maintained and the study was observational. A search of the institutional database using the International Classification of Diseases (9^th^ revision, code 572.0) identified 333 PLA patients diagnosed (January 2004 and March 2010) during hospitalization. PLA was diagnosed if at least 1 of the following criteria was met: (1) pus was drained by percutaneous catheter drainage (PCD); (2) bacterial pathogens were isolated from blood or abscess cultures; and (3) the intrahepatic cavity was resolved with antibiotics, surgery, or PCD. Patients with hemoglobin diseases (HbS, HbC, HbF, and HbE) that could interfere with HbA_1c_ assay, those with conditions influencing red cell turnover (i.e., hemolytic anemia, chronic malaria, or major blood loss), and those infected with pathogens originating from amebiasis were excluded.

### Methods

Two authors retrospectively reviewed the medical records of PLA patients for age, gender, HbA_1C_ levels_,_ clinical presentation, underlying diseases, MDCT parameters, laboratory data at initial presentation, adverse outcomes, and hospital stay duration. The patients were initially divided into 2 groups (diabetic and nondiabetic). Diabetes was considered present if a patient had a hospital discharge diagnosis of type 1 or type 2 diabetes, and/or at least 1 prescription for insulin or an oral antidiabetic agent, and/or had an HbA_1c_ level ≥6.5% in the preceding 2 months [8].

The diabetic patients were further divided into 3 subgroups on the basis of HbA_1C_ levels as follows: HbA_1C_ ≤7% indicating good chronic glycemic control, 7%<HbA_1C_ <9% indicating suboptimal chronic glycemic control, and HbA_1C_ ≥9% indicating poor chronic glycemic control. The HbA_1C_ cut-off value selection was based on previous studies [14]–[17]. The included patients were treated with antibiotics alone, antibiotics (most commonly ceftriaxone) plus PCD, or antibiotics plus surgery. The antibiotic was subsequently adjusted according to the blood or abscess culture results. Pus was aspirated by PCD and sent for microbiological analysis. The adverse outcomes included mortality; metastatic infection (defined as a distal infection with the same bacterium as the PLA culture); septic shock (defined and treated by the criteria of the Surviving Sepsis Campaign) [18]; acute hepatic failure (defined as the development of severe acute liver injury with coagulopathy and encephalopathy); acute respiratory failure (defined as patients requiring mechanical ventilation); upper gastrointestinal (UGI) bleeding (defined as endoscopic evidence of stress-related mucosal bleeding, bright red blood per nasogastric tube, or melena); acute renal failure (defined as an increase in serum creatinine >0.5 mg/dL from baseline) during the hospital stay; acute myocardial infarction (an episode of acute myocardial infarction) during hospitalization, and empyema.

### HbA1C and Glucose Measurements

HbA_1C_ levels that were determined in patients within 1 week prior to their current visit or in the 1^st^ week of admission were collected. HbA_1C_ was analyzed at Tri-Service General Hospital (Taipei, Republic of China) using a blood analyzer (Primus CLC 385; Primus Corporation, Kansas City, MO, USA). This assay used a high-performance liquid chromatography system, standardized to the Diabetes Control and Complication Trial. The laboratory received National Glycohemoglobin Standardization Program Level 1 certification for this method.

The admission glucose level was defined as the plasma glucose level measured at the initial presentation. Hyperglycemia was defined as admission glucose level ≥200 mg/dL, which was used by several previous hyperglycemia studies [2], [3], [19]. The eAG over the previous 3 months was calculated by the equation: AG = 28.7 × HbA_1C_ − 46.7 [9]. The glycemic gap was defined as admission glucose levels obtained at initial presentation minus eAG.

### Statistical Analysis

Continuous data were expressed as means ± standard deviations and analyzed using the two-tailed Student’s *t*-test. Categorical data were expressed as frequencies (%) and tested using the chi-squared or Fisher’s exact test. One-way analysis of variance was used to examine the significance levels for various characteristics, laboratory data, and clinical outcomes in HbA_1C_-categorized groups. Hypoglycemic (blood glucose level <70 mg/dL) diabetic patients were excluded while analyzing the glycemic gap. A receiver operator characteristic (ROC) curve was plotted to classify adverse outcomes in diabetic PLA patients on the basis of the glycemic gap. Univariate logistic regression analysis was used to determine possible predictors associated with adverse outcomes. We selected all parameters for which the *P*-value was <.05 in the initial univariate results and gender, age, and gas formation for multivariate analyses to determine the predictors most strongly associated with adverse outcomes. The data were analyzed using the Statistical Package for the Social Sciences version 17.0 statistical software (SPSS, Inc., Chicago, IL, USA), and differences with *P*-values <.05 were considered statistically significant.

## Results

### The Demographic Data and Underlying Disease

A total of 333 PLA patients were enrolled, but 3 were excluded because of amebic liver abscesses and 1 patient was excluded because of PCD-associated hemorrhagic shock. There were 165 (50.2%) patients with diabetes and 164 (49.8%) patients without diabetes. Among the diabetic patients, 135 (81.8%) had recorded HbA_1C_ levels. Based on the HbA_1C_ levels of the diabetic patients, 33 patients (24.4%) had good glycemic control, 39 (28.9%) had suboptimal glycemic control, and 63 (46.7%) had poor glycemic control. The diabetic patients were older (*P* = .002), had lower incidences of cancer (*P* = .01) and chronic hepatitis B (*P* = .02), and 12.4% lower chance of initially presenting with right upper quadrant (RUQ) abdominal pain (*P* = .02) than the nondiabetic patients (Table 1). The diabetic patients with poor glycemic control were younger than those with suboptimal and good glycemic control (*P* = .02).

**Table 1:** The comparison of the characteristic and underlying disease between non-diabetes mellitus (non-DM) and diabetic mellitus (DM) patients with pyogenic liver abscess.

					Patients with known prior history of DM			
	Non-DM (n = 164 )	DM (n = 165)		*p* value	HbA_1C_ ≦ 7% (n = 33 )	7%<HbA_1C_<9% (n = 39)	HbA_1C_≧ 9% (n = 63 )	*p* value
Age (years)	58.6±17.2	64.2±14.0		.002	66.8±14.5	66.6±13.0	60.1±14.0	.02
Body-mass index	24.1±4.1	25.1±3.7		.12	25.1±3.7	25.5±4.1	33.5±51.3	.59
Male	102(62.2)	90(54.5)		.20	16(48.5)	18(46.2)	41(64.1)	.14
***Underlying disease***								
Biliary tract disease	38(23.2)	40(24.2)		.82	6(18.2)	11(28.2)	13(20.3)	.53
Cancer	37(22.6)	20(12.1)		.01	3(9.1)	5(12.8)	8(12.5)	.86
CHF	1(0.6)	6(3.6)		.06	1(3.0)	1(2.6)	3(4.7)	.84
Chronic hepatitis B	18(11.0)	7(4.2)		.02	1(3.0)	1(2.6)	2(3.1)	.98
Chronic hepatitis C	4(2.4)	1(0.6)		.17	1(3.0)	0(0)	0(0)	.21
Liver cirrhosis	10(6.1)	6(3.6)		.29	1(3.0)	2(5.1)	0(0)	.21
Alcoholism	12(7.3)	6(3.6)		.21	1(3.0)	2(5.1)	2(3.1)	.48
remia	7(4.3)	11(6.7)		.34	1(3.0)	4(10.3)	3(4.7)	.37
CVA	9(5.5)	16(9.7)		.15	3(9.1)	4(10.3)	3(4.7)	.52
***Initial presentation***								
RUQ pain	74(45.1)	54(32.7)		.02	7(21.2)	17(43.6)	19(29.7)	.36
Fever	151(92.1)	146(88.5)		.27	29(87.9)	36(92.3)	54(84.4)	.50

Values are expressed as mean± SD or number (%); CHF, congestic heart failure; CVA, cerebrovascular accident with bed ridden; RUQ, right upper quadrant.

### Laboratory Data and MDCT Parameters

*Klebsiella pneumoniae* (KP) was the most common organism isolated (68.7%) from both blood and abscess cultures of PLA patients. KP infections were more frequently observed in diabetic patients (74.5%) than in nondiabetic patients (62.8%, *P* = .02). In addition, the poor glycemic control group had a significantly higher KP infection rate (89.1%) than the suboptimal (71.8%) and good (63.6%) glycemic control groups (*P* = .03) (Table 2). Triglyceride levels were significantly higher and the sodium levels were significantly lower in the diabetic patients compared with the nondiabetic patients, but there were no significant differences among the glycemic control groups. The albumin levels were significantly lower in the poor glycemic control group than the suboptimal and good glycemic control groups (*P*<.001). The admission glucose levels were significantly higher in diabetic patients than nondiabetic patients (*P*<.001); it was significantly higher in the poor glycemic control group than the suboptimal and good glycemic control groups (*P*<.001). With regard to MDCT for patients with PLA, diabetic patients had significantly higher incidences of ill-defined abscess margins and solitary abscesses than the nondiabetic patients.

**Table 2:** The comparison of the laboratory data and MDCT parameters between non-diabetes mellitus (non-DM) and diabetic mellitus (DM) patients with pyogenic liver abscess.

				Patients with known prior history of DM			
	Non-DM (n = 164)	DM (n = 165)	*p* value	HbA_1C_ ≦ 7% (n = 33 )	7%<HbA_1C_<9% (n = 39)	HbA_1C_≧ 9% (n = 63 )	*p* value
WBC (µL)	14090±6589	14585±6112	.48	14137±4724	14961±6471	14070±5922	.73
*K.P* infection	103(62.8)	123(74.5)	.02	21(63.6)	28(71.8)	57(89.1)	.03
Hemoglobin (g/dL)	12.04±2.16	11.92±2.00	.61	12.08±1.99	11.68±2.17	12.25±1.93	.39
CRP (mg/dl)	21.2±10.0	20.8±10.5	.68	17.5±9.1	22.2±10.8	21.8±10.6	.10
Na(mmol/L)	134.1±10.8	131.8±7.1	.03	133.5±5.9	132.0±3.9	130.5±9.4	.18
Creatinine (mg/dL)	2.08±8.74	1.28±1.19	.25	1.04±0.82	1.27±1.08	1.25±0.96	.53
Albumin (g/dL)	3.22±2.75	2.80±0.79	.07	3.20±0.50	2.96±0.46	2.57±0.88	<.001
T.bil (mg/dL)	2.76±9.63	1.29±1.73	.06	1.08±0.59	1.82±3.10	1.04±0.94	.10
Triglyceride (mg/dL)	118±66	149±100	.004	121±79	139±91	171±120	.10
Alk-P(IU/dL)	185±158.	165±127	.26	169±163	144±92	165±113	.67
AST (U/L)	113±212	93±143	.32	96±154	66±52	96±139	.45
Glucose (mg/dL)	125±37	275±152	<.001	164±73	255±91	330±132	<.001
Size (cm)	6.1±2.9	6.1±2.6	.85	6.0±2.6	5.8±2.5	6.4±2.7	.44
Gas formation	19(11.6)	28(17.0)	.14	1(3.0)	8(20.5)	14(21.9)	.17
Cystic	115(70.1)	113(68.5)	.09	26(78.8)	25(64.1)	45(70.3)	.39
Septum	92(56.1)	109(66.1)	.10	24(72.7)	26(66.7)	40(62.5)	.59
Margin,well defined	100(61.0)	82(49.7)	.004	20(60.6)	20(51.3)	30(46.9)	.51
Multiple	41(25.0)	29(17.6)	.02	5(15.2)	8(20.5)	11(17.2)	.89

Values are expressed as mean± SD or number (%); *K.P, Klebsiella Pneumonia*; WBC, white blood cell count; CRP, C-reactive protein; T.bil, total bilirubin; Alk-P, Alkaline Phosphatase; AST, aspartate Transaminase.

### Clinical Outcomes

Thirty-six (10.9%) patients were treated with antibiotics alone, 249 (75.7%) patients were treated with antibiotics plus PCD and 44 (13.4%) were treated with antibiotics plus surgery. The hospital stay was significantly longer in the poor glycemic control group than the suboptimal and good control groups (*P* = .03). However, no substantial differences were observed in adverse outcomes between the glycemic control groups. Among the 329 PLA patients, 21 died; this yielded an overall 6.3% PLA mortality rate. The nondiabetic group (8.5%) had a higher mortality rate than the diabetic group (4.2%), without statistically significant differences (Table 3). The most common clinical adverse outcomes were acute respiratory failure (14.0%) and septic shock (13.1%). Only acute hepatic failure was significantly higher in nondiabetic (6.1%) patients than diabetic patients (0.6%, *P* = .006).

**Table 3:** The comparison of the treatment and clinical outcome between non-diabetes mellitus (non-DM) and diabetic mellitus (DM) patients with pyogenic liver abscess.

				Patients with known prior history of DM			
	Non-DM (n = 164 )	DM (n = 165)	*p* value	HbA_1C_ ≦ 7% (n = 33 )	7%<HbA_1C_<9% (n = 39)	HbA_1C_≧ 9% (n = 63 )	*p* value
Percutaneous drainage	122(74.4)	127(77.0)	.49	29(87.9)	29(74.4)	51(79.7)	.39
Operation	23(14.0)	21(12.7)	.57	3(9.1)	3(7.7)	9(14.1)	.46
Vasopressor required inthe ED	16(9.8)	11(6.7)	.41	1(3.0)	4(10.3)	2(3.1)	.06
Hospital stay (days)	22.5±14.1	27.0±26.2	.06	20.5±10.7	23.4±10.0	34.1±37.5	.03
Adverse outcomes	70(42.7)	67(40.6)	.70	11(33.3)	16(41.0)	23(35.9)	.78
Mortality	14(8.5)	7(4.2)	.17	0(0)	0(0)	2(3.1)	.31
Metastatic infection	11(6.7)	14(8.5)	.54	2(6.1)	2(6.1)	3(4.7)	.53
Acute renal failure	18(11.0)	11(6.7)	.16	2(6.1)	1(2.6)	5(7.8)	.54
Acute respiratory failure	19(11.6)	27(16.4)	.21	2(6.1)	5(12.8)	11(17.2)	.30
Acute hepatic failure	10(6.1)	1(0.6)	.006	0(0)	0(0)	0(0)	-
Empyema	3(1.8)	3(1.8)	.99	0(0)	1(2.6)	1(1.6)	.66
UGI bleeding	14(8.5)	11(6.7)	.52	2(6.1)	4(10.3)	3(4.7)	.53
Multi-organ failure	10(6.1)	8(4.8)	.61	0(0)	0(0)	3(4.7)	.17
Acute myocardial infarction	3(1.8)	8(4.8)	.18	1(3.0)	2(5.1)	3(4.7)	.85
Septic shock	21(12.8)	22(13.3)	.88	1(3.0)	3(7.7)	9(14.1)	.19

Values are expressed as mean± SD or number (%); UGI bleeding, upper gastrointestinal bleeding.

### The Gap between Admission Glucose Level and eAG

**Figure 1:**
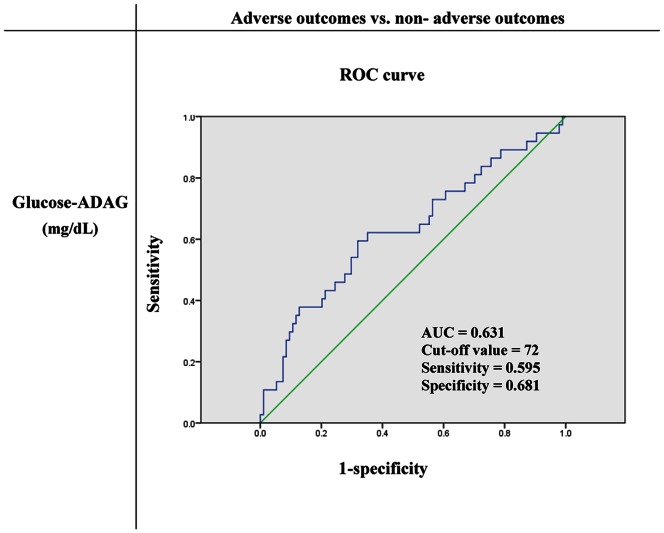
ROC analysis of glycemic gaps to adverse outcomes among diabetic PLA patients. ADAG; A_1C_-derived average glucose AUC; Area under the curve PLA; Pyogenic liver abscess ROC; Receiver operating characteristic.

Three of 165 diabetic PLA patients had no recorded admission glucose levels, and 1 patient with an admission glucose level of 50 mg/dL was excluded from the glycemic gap analysis. The ROC curve revealed that a glycemic gap of 72 mg/dL was the optimal cut-off value for predicting adverse outcomes in diabetic PLA patients. The area under the ROC curve was 0.631 with a sensitivity of 59.5% and specificity of 68.1% (Figure 1).

Compared with diabetic PLA patients with a glycemic gap<72 mg/dL (19.2%), those with glycemic gaps ≥72 mg/dL (41.5%) had a 22.3% relative increase in the incidence of adverse outcomes (*P* = .005), such as higher incidences of metastatic infections, acute respiratory failure, septic shock, empyema, and UGI bleeding. These outcomes did not show statistically significant differences, except for UGI bleeding (*P* = .002) (Table 4).

**Table 4:** Clinical outcome versus Glucose- A_1C_ derived average glucose (ADAG) in diabetic patients with pyogenic liver abscess.

	Glucose-ADAG<72 mg/dL (n = 78 )	Glucose-ADAG≧72 mg/dL (n = 53)	*p* value
Adverse outcomes	15(19.2)	22(41.5)	.005
Metastatic infection	4(5.1)	6(11.3)	.19
Acute renal failure	5(6.4)	3(5.7)	.86
Acute respiratory failure	8(10.3)	10(18.9)	.16
Septic shock	7(9.0)	8(15.1)	.28
Empyema	0(0)	2(3.8)	.08
UGI bleeding	1(1.3)	8(15.1)	.002
Acute myocardial infarction	4(5.1)	2(3.8)	.54
Multi-organ failure	2(2.6)	1(1.9)	.79
Mortality	2(2.6)	1(1.9)	.68
Hospital stay (days)	24.3±17.5	33.0±37.6	.06
Percutaneous drainage	65(83.3)	40(75.5)	.26
Operation	9(11.5)	6(11.3)	.96

Values are expressed as mean± SD or number (%); UGI bleeding, upper gastrointestinal bleeding.

### Univariate and Multivariate Logistic Regression Analysis

Univariate analysis showed that surgery, glycemic gaps ≥72 mg/dL, anemia, creatinine levels >1.3 mg/dl, albumin levels <3.5 g/dL, total bilirubin levels >1.2 mg/dL, and alkaline phosphatase levels >129 mg/dL were correlated with adverse outcomes (*P*<.05), but admission glucose levels >200 mg/dL were not (Table 5). Multivariate analysis showed that only a glycemic gap≥72 mg/dL was a significant predictor of adverse outcomes in diabetic PLA patients.

**Table 5:** Univariate and multivariate logistic regression for risk factors associated with adverse outcomes.

	Univariate analysis	Multivariate analysis
Variable	Odd ratio(95%CI)	Odd ratio(95%CI)
Glucose-ADAG≧72 mg/dL	3.0(1.4–6.5)*	6.9(1.8–26.3)*
Operation	4.5(1.7–11.7)*	2.4 (0.4–13.0)
Age≧65 years	1.04(0.5–2.0)	1.0(0.3–3.5)
Male	0.7(0.4–1.4)	0.9(0.3–3.0)
Anemia^a^	2.0(1.01–4.1)*	0.8(0.2–2.8)
Creatinine >1.3 mg/dL	2.4(1.2–5.1)*	2.3(0.5–9.6)
Albumin<3.5 g/dL	7.0 (1.6–31.1)*	2.2(0.2–21.5)
T.bil>1.2 mg/dL	2.3(1.1–4.6)*	2.3(0.7–7.8)
Alk-P>129 mg/dL	2.2(1.02–4.7)*	1.6(0.5–5.5)
Gas formation	1.7(0.7–3.9)	0.6(0.1–3.0)
Glucose>200 mg/dL	2.2(0.9–4.7)	–

ADAG, A_1C_ derived average glucose;

a Hemoglobin<13 g/dL in men, <12 g/dL in women; T.bil, total bilirubin; Alk-P, alkaline phosphatase; CI, confidence interval.

* *P*<0.05.

Multivariate analysis: included all variable with *P* values <0.05 in the univariate analysis, age≧65 years, male and gas formation.

## Discussion

In present study we have found that: 1) the glycemic gap rather than hyperglycemia on admission was a major predictor of adverse outcomes in diabetic PLA patients; 2); no correlation was observed between chronic glycemic control and adverse outcomes in diabetic PLA patients; and 3) poorer chronic glycemic control were associated with higher incidences of KP infection, hypoalbuminemia, and longer hospital stays in diabetic PLA patients.

Acute hyperglycemia has been linked to increased adverse outcomes in patients with trauma; [19] poorer neurological improvement and symptomatic hemorrhage in r-tPA treated patients with acute ischemic stroke; [20] severity of injury in patients with head trauma; [21] increased risk of in-hospital complications in patients with community-acquired pneumonia; [7], [22] and major adverse cardiac events in patients with acute myocardial infarction [23]. Admission hyperglycemia has been linked with poorer outcomes in patients not known to have diabetes [24]. Nonetheless, admission hyperglycemia in the acute care setting has not consistently been shown to portend a worse prognosis in patients with preexisting diabetes. Furthermore, a recent study revealed that hyperglycemia was strongly associated with increased stays in intensive care units and mortality in critical nondiabetic patients, but not in diabetic patients [6]. Because hyperglycemia is a pronounced feature of diabetes, it is necessarily to consider pre-existing hyperglycemia in diabetic patients when investigating the association between sepsis-induced stress hyperglycemia and adverse outcomes. Our study was the first to investigate whether the glycemic gap was associated with adverse outcomes in diabetic PLA patients. In agreement with other study regarding infectious diseases, [4] we have shown that hyperglycemia on admission could not predict adverse outcomes in diabetic PLA patients. By using the concept of glycemic gap, we were able to eliminate the possible influence of chronic hyperglycemia on acute hyperglycemia by an HbA1c based eAG method. We subsequently found that acute surge of glucose levels beyond the long-term average glucose levels should be regard as a marker for the severity of sepsis in infected diabetic patients, in consistent with the admission hyperglycemia correlates with adverse outcomes in patient without pre-existing diabetes. We observed that elevated glycemic gaps >72 mg/dL can predict adverse outcomes in diabetic PLA patients rather than admission hyperglycemia alone. We hypothesize that the glycemic gap represented the true stress- induced hyperglycemia in infected diabetic patients. An increased stress response may reflect increased severity and consequent higher incidences of adverse outcomes. Egi et al demonstrated that pre-existing glycemic control may alter the association between acute hyperglycemia and mortality in diabetic patients with critical illnesses [25]. We thought that the concept of glycemic gap provide a new insight into the paradox of discordant results regarding the correlation between hyperglycemia and adverse outcomes in acute-ill patients with or without preexisting diabetes.

We used HbA_1C_ levels to categorize a patient’s chronic glycemic control status and found that diabetic PLA patients with poor glycemic control had higher incidences of KP infection, longer hospital stays, hypoalbuminemia, compared with those with suboptimal and good glycemic control. Nevertheless, poorer chronic glycemic control was not associated with adverse outcomes or mortality in the present study. Several studies have demonstrated that KP-PLA frequently occurs in diabetic patients [26]. Poor chronic glycemic control can affect cell-mediated and humoral immune responses, resulting in infection [27]. Diabetic patients are susceptible to KP infections due to neutrophil phagocytosis of K1 and K2 capsular serotypes are impaired by poor glycemic control [28]. KP is the most commonly isolated microorganism in Asian PLA patients and accounts for most septic metastatic infections [11], [29]. Our results further demonstrate that poorer glycemic control were associate with increased incidence of KP-PLA in diabetic patients.

Poor chronic glycemic control has been associated with significantly longer hospital stays for diabetic patients undergoing total joint arthroplasty; [30] increased risk of hospitalization for heart failure with type 2 diabetes; [31] unfavorable neurological and functional outcome of acute ischemic stroke; [32] increased risk of pneumonia related hospitalization; [33] a greater incidence of adverse outcomes and longer hospital stay after cardiac surgery in diabetic patients; [34] and higher risk of major amputation in critical limb ischemia patient undergoing percutaneous transluminal angioplasty [35]. Our study disclosed that diabetic PLA patients with poor chronic glycemic control had longer hospital stays than those with suboptimal and good glycemic control. We speculate that prolonged hospital stays may result from treatments for acute complications such as septic shock, acute respiratory failure, and difficult glucose control during hospitalization. Our results are in consistent with previous studies regarding PLA in diabetic patients had lower rates of cancer and RUQ pain during their initial presentations, compared to nondiabetic patients [36]. Although studies regarding distinctive CT features of PLA between diabetic and nondiabetic patients were limited, previous studies found that ill-defined margins and solitary abscess were more common in KP PLA [37], [38]. Because KP is more commonly as the pathogen in diabetic PLA patients, it is reasonable that diabetic PLA patients have higher incidences of ill-defined solitary abscess. Hypoalbuminemia may reflect the incidences of diabetic nephropathy-related proteinuria and suppressed albumin synthesis due to acute illnesses [39]. The diabetic PLA patients had higher triglyceride levels than non-diabetic PLA patients, probably because diabetes dysregulates lipoprotein metabolism accompanied by insulin resistance [40]. The higher incidence of acute hepatic failure in non-diabetic patients compared with diabetic patients may result from the higher comorbidity of cancers in non-diabetic patients than diabetic patients, especially hepatocellular carcinoma that accounted for approximately half (43.2%) of all cancers in non-diabetic patients.

An unexpected finding in this study was that diabetic PLA patients with glycemic gaps ≥72 mg/dL had higher incidences of UGI bleeding than those with gaps <72 mg/dL during hospitalization. A critical illness can result in stress-induced ulcers resulting from complex pathophysiological mechanisms, including decreased gastric mucosal blood flow, increased gastric mucosal permeability with increased acid back-diffusion, and ischemia–reperfusion injuries [41]. Acute Physiology and Chronic Health Evaluation (APACHE) II scores were independent predictors of acute gastrointestinal bleeding, i.e., the incidence of gastrointestinal bleeding was 1.45 times higher for every 10 point increase in the APACHE II score in patients with acute renal failure [42]. A positive correlation between plasma cortisol levels on admission and stress-induced ulcers have been demonstrated in patients with acute severe head injuries [43]. We speculate that the association between elevated glycemic gaps and UGI bleeding might have reflected greater severity of illness and physiological stress that promoted to the development of stress-induced ulcers with bleeding. However, further research is needed to discern whether a glycemic gap can predict the need of prophylaxis for stress-induced ulcers in diabetic PLA patients and in other settings.

Our study had several limitations. First, it was retrospective and selection bias could be existed. Second, we did not specifically address the treatments for hyperglycemia and glycemic controls during hospitalization although the adequacy of glycemic control during hospitalization may have influenced the outcomes [24]. Third, there were 30 (18.2%) diabetic patients without recorded HbA_1C_ levels, including 5 patients who died. Our results may not be generalized to all diabetic PLA patients. Hence, further prospective and controlled studies are needed.

### Conclusion

This study demonstrated that an elevated glycemic gap, rather than admission hyperglycemia or chronic glycemic control, was correlated with adverse outcomes in diabetic PLA patients. Poorer glycemic control in diabetic PLA patients resulted in higher incidences of KP-PLA and longer hospital stays. The concept of glycemic gap warrants further large-scale prospective studies in other acute illness in patients with diabetes.
